# Pain in castration-resistant prostate cancer with bone metastases: a qualitative study

**DOI:** 10.1186/1477-7525-9-88

**Published:** 2011-10-12

**Authors:** Adam Gater, Linda Abetz-Webb, Clare Battersby, Bhash Parasuraman, Stuart McIntosh, Faith Nathan, Elisabeth C Piault

**Affiliations:** 1 Mapi Values, Bollington, Cheshire, UK; 2 AstraZeneca, Alderley Park, Cheshire, UK; 3 AstraZeneca LP, Wilmington, Delaware, USA; 4 Mapi Values, Boston, Massachusetts, USA

## Abstract

**Background:**

Bone metastases are a common painful and debilitating consequence of castration-resistant prostate cancer (CPRC). Bone pain may predict patients' prognosis and there is a need to further explore CRPC patients' experiences of bone pain in the overall context of disease pathology. Due to the subjective nature of pain, assessments of pain severity, onset and progression are reliant on patient assessment. Patient reported outcome (PRO) measures, therefore, are commonly used as key endpoints for evaluating the efficacy of CRPC treatments. Evidence of the content validity of leading PRO measures of pain severity used in CRPC clinical trials is, however, limited.

**Methods:**

To document patients' experience of CRPC symptoms including pain, and their impact on health-related quality of life (HRQL), semi-structured in-depth qualitative interviews were conducted with 17 patients with CRPC and bone metastases. The content validity of the Present Pain Intensity (PPI) scale from the McGill Pain Questionnaire (MPQ), and the 'Average Pain' and 'Worst Pain' items of the Brief Pain Inventory Short-Form (BPI-SF) was also assessed.

**Results:**

Patients with CRPC and bone metastases present with a constellation of symptoms that can have a profound effect on HRQL. For patients in this study, bone pain was the most prominent and debilitating symptom associated with their condition. Bone pain was chronic and, despite being generally well-managed by analgesic medication, instances of breakthrough cancer pain (BTcP) were common. Cognitive debriefing of the selected PRO measures of pain severity highlighted difficulties among patients in understanding the verbal response scale (VRS) of the MPQ PPI scale. There were also some inconsistencies in the way in which the BPI-SF 'Average Pain' item was interpreted by patients. In contrast, the BPI-SF 'Worst Pain' item was well understood and interpreted consistently among patients.

**Conclusions:**

Study findings support the importance of PRO measures of pain severity as key endpoints for evaluating the efficacy of treatments for CRPC, particularly for patients with bone metastases where episodes of BTcP are common. Qualitative evidence from CRPC patients supports the content validity of the BPI-SF ''Worst Pain' item and promotes use of this item for measuring pain severity in this population.

## Background

Prostate cancer is the second most common cancer in men with a worldwide age-standardised incidence rate (ASR) of 28.1 per 100,000 [1]. It is often a slow-growing cancer but, despite treatment, spread of cancerous cells to other sites in the body occurs frequently [2]. Hormonal therapy in the form of androgen blockade/suppression can limit disease progression in patients with advanced metastatic prostate cancer, but many patients become resistant to such therapy within 1.5-3.0 years of commencing treatment [3]. The development of castration-resistant prostate cancer (CRPC) is associated with rapid disease progression, such that survival rarely exceeds 9-12 months [4]. Indeed, almost all deaths resulting from prostate cancer can be attributed to castration-resistant disease [5].

Bone metastases occur in more than 90% of men with CRPC [6] and, as a result of tumor deconstruction of bone and the compromise of nearby nerves, many patients experience considerable pain [7]. Furthermore, bone metastases may lead to skeletal-related events, such as hypercalcaemia bone fractures and spinal-cord compression, which can also increase pain and may impair patients' physical functioning and health-related quality of life (HRQL) [8-10].

Pain has been identified as an important indicator of overall survival in men with metastatic CRPC [11,12]. Despite the existence of guidelines for cancer pain management, pain is often under treated in cancer patients [13,14], which may in part be because of the difficulties in assessing pain in a valid and reliable manner. Physicians often underestimate patients' experience of pain [15] and, in the absence of a biomarker for pain assessment, patient reported outcome (PRO) measures have become the dominant outcome measures in pain assessment as they reflect the inherently subjective nature of pain [16].

While it is widely recognized that pain is a multi-dimensional construct [17], a unidimensional approach centered on regular assessments of pain severity via PRO measures been adopted in chronic pain trials for many years [16]. Pain severity is frequently the most important contribution to cancer patients' experience of pain, as demonstrated by its interference with quality of life and daily functioning [15,18]. As such, assessment of pain severity by short, user-friendly measures administered on a regular basis, allows cancer patients' experiences of the evolution of pain to be captured and quantified.

Two of the most commonly implemented self-completed measures of pain severity in clinical research are the McGill Pain Questionnaire Short-Form (MPQ-SF) [19,20] and The Brief Pain Inventory Short-Form (BPI-SF) [21]. The MPQ-SF measures the sensory, affective, and evaluative components of pain and comprises 15 items, including a 6-point verbal rating scale (VRS) asking patients to rate their present pain intensity (PPI) using one of six descriptors (0 - No pain, 1 - Mild, 2 - Discomforting, 3 - Distressing, 4 - Horrible, 5 - Excruciating). The BPI-SF assesses pain severity (four items) and pain interference (11 items). Pain severity is measured through patients' rating of their level of current pain, average pain, least pain in the last 24 hours and worst pain in the last 24 hours, using an 11-point numerical rating scale (NRS) ranging from 0 (no pain) to 10 (pain as bad as you can imagine). Although these four items can be used to form a composite score of pain intensity, the 'Worst Pain' and 'Average Pain' items of the BPI-SF are often used in clinical trials to singly represent pain severity [21] as supported by the Initiative on Methods, Measurement, and Pain Assessment in Clinical Trials (IMMPACT) [16,22,23].

When selecting a PRO measure for use in clinical trials to document treatment benefit, it is necessary to demonstrate that the selected measure is fit for its intended use in a specific context of use [24]. A key consideration in this regard is evidence of content validity (i.e. that the measure captures concepts that are of importance and clinical relevance to patients and in a manner that is comprehensive, relevant and comprehensible to patients) [24,25]. Documentation of the content validity of a measure is specific to its context of use, including but not limited to the population of interest, and is typically established through the generation of evidence from qualitative research comprising patients whose clinical and demographic characteristics are similar to those of the patients enrolled in the respective clinical trials.

Recent research has provided insight into the experiences of patients with CRPC, however there is a need to further understand the experiences of CRPC patients with bone metastases, particularly with regard to patients' experience of pain [26,27]. Furthermore, while patients experiencing pain were involved in the development of both the MPQ and BPI-SF, there is currently no available evidence of the content validity of these measures for use in CRPC patients with bone metastases.

The present study, therefore, had two main aims: 1.) To investigate, via qualitative research, the experiences of patients with CRPC and bone metastases, particularly in regards to patient experiences of bone pain and resulting HRQL impact 2.) To assess the content validity of the MPQ-SF PPI and the BPI-SF 'Average Pain' and 'Worst Pain' items within this population.

## Methods

### Study design

Face-to-face in-depth qualitative interviews were conducted with 17 patients with CRPC and confirmed bone metastases. A combined approach was taken whereby the interviews included a detailed concept elicitation phase followed by thorough cognitive debriefing of the selected pain measures.

### Patient recruitment

Patients eligible for participation in the study were recruited in the United States with the help of clinicians from two clinical practices and advertisements from a commercial recruitment agency. To be eligible for participation in the study, patients had to have a clinician-confirmed diagnosis of CRPC with documented evidence of bone metastases (CRPC M1) and be at least 18 years or age. Participants were also expected to be currently experiencing bone pain or to have experienced bone pain recently. Exclusion criteria included evidence of central nervous system metastases, severe or uncontrolled systemic disease, or any other significant clinical disorder.

### Ethics

The study was conducted in accordance with the Declaration of Helsinki, and was approved by an Independent Review Board. Written informed consent was obtained from all patients prior to entry into the study.

### Interview methods

All interviews were conducted by trained interviewers using a semi-structured interview guide. The interview guide was informed by consideration of prostate cancer literature as well as published articles documenting the development and validation of the MPQ-SF and BPI-SF. During the initial 'concept-elicitation' phase of the interview, patients were asked a series of open-ended questions (e.g. could you describe for me your experiences of prostate cancer?) designed to investigate their experience of symptoms related to CRPC and bone metastases (with particular focus on pain). At this stage, care was taken to avoid the use of leading questions, in order to ensure that patients' spontaneous responses were captured.

In the second 'cognitive debriefing' phase of the interview, patients were asked to complete the MPQ-SF PPI, and the BPI-SF Average Pain and Worst Pain items as part of a 'think-aloud' exercise in which patients were encouraged to vocalize their decision making process when selecting response choices for each of the measures. Patients were then asked detailed questions designed to verify the relevance and level of clarity (i.e. absence of ambiguity, understanding of terms) of the measures, and to confirm that the items and response options measured pain intensity adequately (e.g. in your own words what is this question asking? How did you decide which answer to give?). Patients were also asked to identify which of the three PRO measures best documented their experience of bone pain. Again the line of questioning was designed so to not lead or bias patient responses.

A pilot interview was conducted with one patient and, based on that patient's feedback, the interview guide was shortened and refocused. An interim analysis of the data collected from this interview and the subsequent 10 interviews was also conducted (Round I), after which the interview guide was revised to incorporate additional questions designed to further explore the terminology that patients used to describe the pain that they experienced and patient understanding and interpretation of the selected PRO measures in the final 6 interviews (Round II).

### Qualitative analysis

All interviews were audio-taped and transcribed verbatim for the purpose of qualitative analysis. Written interview transcripts were then entered into a qualitative software package (Atlas.Ti) which was used to facilitate the analysis of interview transcripts.

Patient responses during the open-ended concept elicitation phase of the interview were analysed using a Grounded Theory approach whereby sections of transcripts from individual patients (i.e. quotes) were assigned codes reflective of underlying concepts [28,29]. In this approach, the meaning of concepts are discovered via the words and actions of participants from the ground up, rather than from application of a *priori *theory or understanding [30]. This approach is particularly useful where, as here, the intention is to build an overall picture of patients' experience and to understand the way in which elicited concepts interrelate with one another. In contrast, qualitative analysis of patient responses during 'cognitive debriefing' focussed specifically on whether the concepts and items comprising the selected PRO measures were relevant, appropriate and understood by patients in the way in which the developers had originally intended [24].

Interview transcripts were coded and analysed by analysts trained in qualitative analysis techniques. To ensure consistency among analysts, a provisional analysis of the first three transcripts in Round I was conducted by all analysts and a reliable coding scheme was then developed based on consensus among analysts. Although the primary purpose of qualitative research is not to assess concept frequency, a count of the number of patients who mentioned a given concept at least once during an interview was recorded during the process of analysis in order to provide an indicator of the relative importance of each concept within the study sample.

There exist no definitive guidelines regarding recommended sample sizes for qualitative studies and available guidance actually states that the number of patients is not as critical as interview quality, with sample size depending on the completeness of information obtained from the analysis of interview transcripts [24]. That the concepts elicited by patients had been fully explored during the interviews was assessed by confirmation of conceptual saturation. Saturation is defined as the point at which no new relevant or important information emerges with the collection of more data [24,28,31] and recent investigations suggest that conceptual saturation is typically achieved within 12-13 interviews [31,32]. Sample sizes of this magnitude are also considered to be generally acceptable for confirmation of user understanding of PRO measures via cognitive debriefing.

## Results

### Patient Demographic and Clinical Characteristics

Seventeen men with CRPC participated in this qualitative research study. The average age of men participating in the study was 71 years (range 53-86) and all but one participant were Caucasian. On average, participants had been diagnosed with prostate cancer for 7 years and had bone metastases for 1.7 years. The sample included patients with well differentiated, moderately differentiated and poorly differentiated or undifferentiated cancer, as defined by Gleason scores [33]. All patients were receiving medication for pain relief and only two patients (12%) reported experiencing more than moderate bone pain, as assessed by ratings on a 5-point Likert-type scale (very mild - very severe) completed on the day of the interview. Demographic and clinical characteristics of the sample are displayed in Table 1.

**Table 1:** Patient demographic and clinical characteristics (n = 17)

Characteristic	
Age in years, mean [range]	71.1 [53 - 86]

Mean years since prostate cancer diagnosis	7.0

Mean years since onset of bone metastases	1.7

Cancer stage*	

Well differentiated (Gleason score 2-4)	2 (12%)

Moderately differentiated (Gleason score 5-7)	5 (29%)

Poorly differentiated or undifferentiated (Gleason score 8-10)	5 (29%)

Patient-rated overall bone pain**	

Very mild	3 (18%)

Mild	3 (18%)

Moderate	8 (47%)

Severe	2 (12%)

Very severe	0

Pain relief medication ***	

Ibuprofen	3 (18%)

Percocet^® ^(acetaminophen; oxycodone hydrochloride)	8 (47%)

Lortab^® ^(acetaminophen, hydrocodone bitartrate)	1 (6%)

Oxycontin^® ^(oxycodone hydrochloride)	5 (29%)

Tylenol^® ^(acetaminophen)	4 (24%)

Zometa^® ^(Zoledronic acid)	2 (12%)

Vicodin^® ^(acetominophen, hydrocodone bitartrate)	1 (6%)

Darvocet^® ^(acetominiphen; propoxyphen hydrocholoride)	1 (6%)

*Missing data (n = 4) ** Missing data (n = 1) *** Patient could be receiving multiple pain relief medications

### Concept Elicitation Phase: Patients' experiences of CRPC and bone metastases

Patients' experiences of bone metastases associated with CRPC and/or its treatment comprised a constellation of symptoms of which bone pain, fatigue and low energy were predominant. CRPC localized symptoms manifested as urinary-related symptoms such as increased urinary frequency, urinary incontinence, pain upon urinating and difficulties initiating or maintaining urinary flow. Difficulties in getting or maintaining an erection were also reported by some patients. Further issues mentioned by patients included gastrointestinal disturbances (such as constipation, diarrhea, bloating and nausea/vomiting), hot flashes (fever/sweats/chills) and changes in taste perception, appetite and weight. However, these issues were largely attributed by patients as being side effects of their current treatment regimens.

Consideration of qualitative data obtained during the interviews revealed that no new concepts were elicited as data collection neared completion (Table 2). All concepts were first elicited during round I of the interviews, with approximately 85% of concepts elicited within the first five interviews. This suggests that conceptual saturation of CRPC-related signs and symptoms was achieved within this sample.

**Table 2:** Patient-reported signs and symptoms of CRPC

Concept	Round I											Round II						Concept Frequency (%)
			
	PT 01	PT 02	PT 03	PT 04	PT 05	PT 06	PT 07	PT 08	PT 09	PT 10	PT 11	PT 12	PT 13	PT 14	PT 15	PT 16	PT 17	
**Bone Pain**		**X**	X	X	X	X	X	X	X	X	X	X	X	X	X	X	X	16 (94)

BTcP (incident)				**X**		X			X				X		X			5 (29)

BTcP (idiopathic)										**X**	X		X				X	4 (24)

BTcP ("end-of-dose failure")			**X**	X		X			X		X	X					X	7 (41)

**Skeletal-related events (fractures)**		**X**							X									2 (12)

**Fatigue**	**X**		X		X	X		X	X	X	X							8 (47)

**Low energy**	**X**	X		X		X	X	X		X								7 (41)

**Loss of strength**		**X**					X						X					3 (18)

**Urinary dysfunction**	**X**	X		X	X	X	X	X	X	X	X	X					X	12 (71)

Blood in urine							**X**			X		X						3 (18)

Can't empty bladder				**X**			X											2 (12)

Increased urinary frequency during the day		**X**		X		X											X	4 (24)

Difficulty starting urination				**X**			X				X							3 (18)

Urinary incontinence				**X**			X		X		X							4 (24)

Weak or interrupted flow of urine				**X**	X		X		X									4 (24)

Increased urinary frequency during the night	**X**			X		X												3 (18)

Painful or burning urination							**X**	X										2 (12)

Poor stream				**X**	X													2 (12)

**Erectile dysfunction**									**X**				X					2 (12)

**Fever/sweats/chills**		**X**	X				X	X					X			X		6 (35)

**Numbness/loss of sensation**						**X**	X	X							X			4 (24)

**Altered taste perception**	**X**														X			2 (12)

**Loss of appetite**						**X**					X				X			3 (18)

**Weight loss**	**X**								X		X							3 (18)

**Weight gain**		**X**				X												2 (12)

**Gastrointestinal disturbance**			**X**	X	X	X	X		X		X							7 (41)

Bloating				**X**		X												2 (12)

Constipation				**X**	X	X					X							4 (24)

Diarrhea			**X**			X												2 (12)

Nausea/vomiting				**X**		X	X		X		X							5 (29)

**Anxiety**				**X**							X							2 (12)

**Depression**				**X**			X				X		X					4 (24)

Note: **X **indicates the first time that the respective concept was elicited by a participant.

In accordance with main study aims, patients' experiences of bone pain were thoroughly explored. This symptom was spontaneously mentioned by 16/17 patients during open-ended discussion which highlights the importance of this concept for patients. Feedback from patients during these interviews suggests that patients were able to distinguish pain caused by bone metastases from other types of pain based not only on the location of the pain but also the intensity and temporal features of the pain including onset, frequency and duration: *'The pain that I have, it's a really different pain. And I've had pain before in my life. But it's so different, because it's very intense. It's very severe. It really hurts'*. Bone pain was often localized in the lower back or 'tailbone'. Other sources of bone pain were synovial joints (including knees, hips and shoulders) and the ribs and neck. Whilst bone pain was the predominant form of pain reported by patients, some patients currently receiving treatment via chemotherapy also referred to pain accompanied by feelings of numbness and loss of sensation, particularly in extremities such as the feet: *"And I've had trouble with my feet - they tend to be a little bit on the numb side, and hopefully that will dissipate"*. It is likely that this is indicative of chemotherapy-induced peripheral neuropathy (CIPN), a common side-effect associated with neurotoxic chemotherapy drugs [34].

Patients reported that the pain they experienced was manageable with analgesic medication such as acetaminophen and opioids, but never completely goes away: '*Well, I control it somewhat with this medication that I'm taking, so that has a lot to do with how I could say it feels'; 'The more medications I take, the lighter the pain will be'; *'*I have not had one solid day of relief without pain whatsoever'*. Furthermore, despite management by analgesic medication, patients did experience some variation in the level of pain experienced which was suggestive of breakthrough cancer pain (BTcP): *'Mine varies quite a bit. It goes from hardly any pain at all to severe'; 'And it's constant... it's just sometimes worse than other times'*. BTcP is defined as a "transient exacerbation of pain that occurs either spontaneously or in relation to a specific predictable or unpredictable trigger despite relatively stable and adequately controlled background pain" and is typically classified as: 1.) incident or provoked; 2.) idiopathic or spontaneous; 3.) "end-of-dose failure" of a long acting opioid [35,36]. Consistent with these definitions, 9/17 patients (53%) reported experiencing BTcP.

Pain for some patients (n = 5) was exacerbated by chemotherapy, and physical activities such as gardening, walking or standing: '*After I went through chemotherapy on Tuesday, the third day after chemo,...every bone in my body from my ankles to my head was in pain and there was no medication or any comfort that I had or had access to that could make that pain go away. It actually kept me bedridden for 3 days*.'; *'If you're trying to do a whole lot, then you probably have a whole lot of pain. If you're not trying to do anything, you're not going to have much pain. Or you're only going to have some, but you're not going to be as bad'*. In contrast, for other patients (n = 4) there was no apparent cause for episodes of exacerbated pain: *'I would say I just got up in the morning, the pain was there. It was like a severe charley horse and I just couldn't make it go away. I increased my medication. I took a stronger oxycodone, I took a 20, because that will usually bring down my pain level, but that didn't even affect it either. It was just there with me the whole day and gradually, I would say over a period of 24 to 36 hours, it finally kind of worked itself out so I could kind of walk around and live with it again'*.

End-of-dose failure, defined as pain that occurs at the end of the timeframe in which pain medication is intended to be effective, was commonly experienced by patients (n = 7). Such pain was frequently responsible for waking patients from their sleep: *'And it gives me some relief maybe where I can drop off and sleep a little more. And then in a couple of hours it's back.'; '...what I was doing helped a little while, but didn't give me relief for very long'*. Delays in obtaining pain relief after taking pain medication also impaired patients' activities the next morning: *'...but it takes me an hour or so to get things limbered up in the mornings in there and my drugs that I take in there to kick in.'*

The pain associated with bone metastases had debilitating consequences on patients' lives with patients reporting impairments in daily functioning from a physical, emotional and social perspective. The physical impact of bone pain typically manifested in patients' experiences of difficulties with walking or even standing: *'The worst part is the relationship between movement and function and this pain. Because when I just try to do anything, I can get up out of the chair, anything like this - I know it's going to hurt. And it usually hurts, and so I'll try to minimize it as much as I can'*. Patients reported that the experience of bone pain and associated limitations in physical functioning meant that their ability to perform their daily activities and to engage in their usual social activities had been affected: '*[Bone pain] prevents me from doing many things that I would do, or would like to do, and normally would do, but I don't do*.'; '*I can't seem to do things that I've always done for myself and things that I like to do for other people...at the present time, it's more of a burden to me than at any time in my life. Right now pain is affecting my daily lifestyle*.'

Further to these physical impairments, patients reported being unable to sleep, feeling tired, and feeling irritable because of bone pain. Patients also described feeling depressed, anxious and stressed, particularly in relation to BTcP when it was evident that pain was getting worse: '*The pain can immobilize you, where it brings on depression. It brings on fear. It brings on anxiety. It brings doubt...stress*.'; '*When you have pain that strong, you can't help but have some fears and worries about it'*.

**Figure 1:**
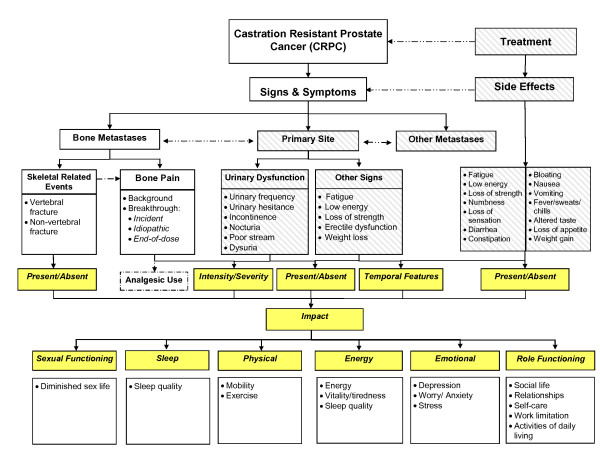
**Disease model of patients' experience of CRPC with bone metastases**.

Based on collective feedback from patients, a disease model outlining patients' experiences of living with CRPC with bone metastases in the context of disease pathology was developed (Figure 1).

### Cognitive Debriefing Phase: Patients' understanding and differentiation of current, worst and average pain

Responses to open-ended questions regarding the perception of 'Current Pain', 'Worst Pain' and 'Average Pain' revealed that patients were able to distinguish between these three distinct concepts. Patients talked about 'Current Pain' as representing the intensity of their pain 'right now' whereas 'Worst Pain' represented the highest intensity of their pain within a given time frame. Patients, however, provided two alternate explanations of 'Average Pain'. For example, some patients considered 'average pain' to represent a value in-between the most and least severe pain that they had experienced over a given time frame, while others described 'average pain' as representing the level of pain experienced 'most of the time'.

### MPQ-PPI item

**Figure 2:**
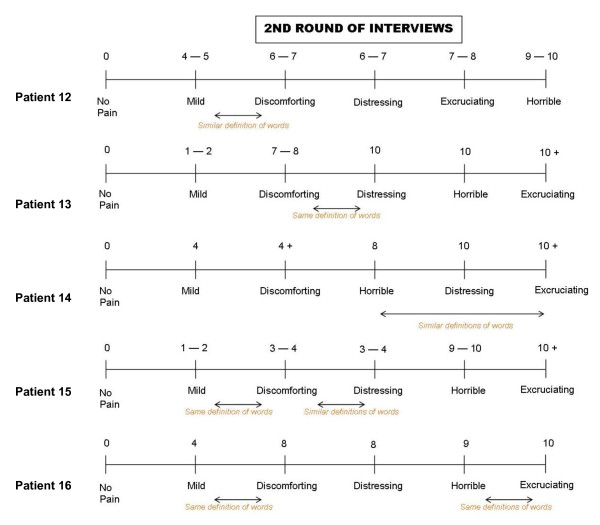
**Patients' representation and rating of the descriptors from the McGill Pain Questionnaire-- Present Pain Intensity (Round II)**.

Most of the patients interviewed did not experience any difficulty in understanding the term "current pain intensity", although it was evident from the responses of three participants that they were not thinking about their "current pain intensity" when answering this question but were thinking back to their worst pain in the past week or the past 24 hours: '*Well I was thinking that that was when it hurt at its worst, perhaps, not necessarily now it was hurting at this particular moment, but how it hurts at its worst time*'. Hardly any difficulties were reported when patients were asked how hard it was to select an appropriate response option for this item. However, some patients had difficulty understanding the meaning of the pain descriptors (no pain, mild, discomforting, distressing, horrible, and excruciating) and the relation of these descriptors to the bone pain they experienced. Patients also found it difficult to differentiate between the pain descriptors used by the MPQ-PPI, with some descriptors seen to be very similar, if not synonymous: '*Yeah, the words mild and discomforting - what's the difference between those? And then discomforting and distressing? Distressing is more of a psychological thing, I would think, than a measurement of pain. Horrible is a fear type thing instead of a measurement of pain. Excruciating - that's probably a pretty accurate measurement of pain. But some of the words there - discomforting, distressing, and horrible I don't care for*.' To further investigate patients' perceptions of the response continuum, as part of the revised interview guide, the final 6 patients interviewed were asked to assign numerical values (from 0-10) to each pain descriptor. Patient answers revealed that there was no standard response continuum and that the distance between individual response options were not interpreted as being equal, as would be expected in a truly linear scale (Figure 2).

### BPI-SF 'Worst Pain' and 'Average Pain' items

Patients appeared to experience very little difficulty understanding the BPI-SF 'Worst Pain' item, correctly interpreting that they were to answer the item by indicating the severity of worst pain experienced in the past 24 hours: *'At the time that my pain was the worst in the last 24 hours, how would I rate that pain as far as everything that I've experienced in my life, from no pain to pain as bad as you can imagine. I would say that my pain the last 24 hours was a seven'*. Patients were asked to define levels of meaningful improvement or worsening in pain using their initial scores as a reference point. New scores provided by all but one patient reflected improvements or worsening in pain where appropriate, with responses indicating that increments or decrements of 2 to 3 points would be considered meaningful.

In deciding on their average pain, 11 patients based their decision on the value between their worst pain and least pain over the specified recall period ('*Well, to me, that's taking the worst pain you had in the last while and the lowest one and kind of average them out over a timeframe*') while 6 patients based their decision on the level of pain that they experienced 'most of the time' ('*I don't know. I guess average is what it is most of the time. And most of the time, when I'm trying to walk or something like that, it's around a five*'). Recall time for average pain varied between patients ('since beginning treatment'; 'last week'; 'last 24 hours') and patients recommended including a clear timeframe for clarity. Again patients indicated that increments or decrements of 2 to 3 points for average pain would be considered meaningful. When asked, patients identified scores of 1-3 as representing an acceptable level of pain, and scores of 7-9 as representing an unacceptable level of pain.

When asked which of the presented items they preferred, one patient had no preference while all other patients preferred the items from the BPI-SF: '*The brief pain inventory, because it's simpler to answer, it's easier to read, and it stands out*.'; '*I like the brief pain inventory better because I can relate to - like I said, I didn't care for the words discomforting, distressing, and horrible*.' Some patients commented that the BPI-SF Worst Pain item was a more accurate reflection of the degree of pain experienced (*'I'm lucky that I have a lot of time that I don't have very much pain, but there are times where I have quite fairly severe pain, so an average really isn't an accurate picture'*), while others preferred the BPI-SF Worst Pain item because it was easier to recall ('*I think three is a little more useful, the one about your worst pain in the last 24 hours, because it's more in your mind, more present in your mind*'). A similar number of patients considered that, because of fluctuating pain levels, the BPI-SF Average Pain item was a better indicator of pain experienced: *'I think average, probably, for the simple reason that I don't think I have that many episodes of really bad pain. I think mine is more on the same level or plane most of the time'*.

## Discussion

Based on qualitative evidence from CPRC patients with bone metastases, a disease model outlining the experiences of such patients in the context of disease pathology has been developed. Such models are valuable in terms of identifying key measurement concepts which can be used to demonstrate treatment benefit, providing insight into how best to measure these particular concepts and providing a contextual basis for interpreting study findings [37]. As evident within this model, among the constellation of symptoms experienced by patients with metastatic CRPC, pain (and specifically bone pain) is of paramount importance. This is consistent with previous qualitative and clinical studies where pain is reported to be the most frequently observed symptom among CRPC patients [26,27]. Findings from this study reveal that pain associated with bone metastases is chronic in nature, but generally well-managed by analgesic medication. Episodes of BTcP, however, are common and particularly debilitating for patients. Patients in the present study reported experiencing worsening of pain in response to mild physical exertion or particular activities of daily living (e.g. walking to the station, standing up), as a result of end of dose failure of analgesic medication and in some instances for no discernable reason at all.

Given the fluctuating nature of pain associated with bone metastases, subjective reporting of pain is time dependent. Multiple reports of pain over time allow integration of symptom evolution in the assessment of patients' pain severity. For repeated use, the PRO measure selected for pain assessment should be short and easy for patients to complete. In this study cognitive debriefing of three single-item measures of pain severity commonly included as endpoints in oncology clinical trials, the MPQ-SF PPI item and 'Average Pain' and 'Worst Pain' items of the BPI-SF, was conducted to determine content validity in patients with CRPC.

Whilst the majority of patients were able to accurately interpret the concept of 'current pain' (assessed by the MPQ-SF PPI item), due to the fluctuating nature of pain experienced by CRPC patients with bone metastases, assessments of 'current pain' may not be the most accurate or informative way of assessing pain severity in this population. Indeed, previous research has highlighted that ratings of current pain, among patients experiencing persistent pain, are often lower than ratings of recalled worst or average pain over a given time-period [38].

The concept of 'worst pain' appeared to be interpreted correctly and consistently among patients. By contrast, however, there appeared to be some variability in the way in which patients interpreted the concept 'average pain'. Without consensus among patients regarding the definition of average pain, it would be difficult to determine whether differences among patients are a true reflection of individual differences in pain experience or a result of variations in interpretation of the concept by patients, ultimately limiting the validity of such assessments. As such, 'worst pain' may be the most appropriate measure of pain severity for use in clinical trials. Furthermore, there is evidence to suggest that ratings of 'worst' pain are more representative of the burden experienced by patients in relation to pain and may be more reliable to report; given that when a patient with persistent pain thinks back to their pain over a period of time, they tend to focus their response on their 'worst pain' even when asked about their 'average pain [38,39]. Reduction of 'worst pain', therefore, can be seen as a key indicator of treatment efficacy for patients.

Feedback from patients regarding the different rating scales adopted by the MPQ-SF PPI (0-6 VRS) and BPI-SF (0-10 NRS) highlighted the superiority of NRS ratings scales for the assessment of pain severity. It appeared to be challenging for patients to define and distinguish between the pain descriptors used in the MPQ-SF PPI scale and there was little or no consensus among patients regarding the grading of each response option. As a result, the MPQ PPI scale could be expected to demonstrate limited sensitivity to changes in the level of pain experienced by patients. Indeed, the limited sensitivity of VRS scales to clinical changes is a commonly held weakness of the use of such measures in pain assessment [40].

By contrast, patients experienced little or no difficulty rating the worst or average pain using a 0-10 NRS. From a measurement perspective, the use of an 11-point NRS scale to measure pain severity provides a simple, non-burdensome response format that is likely to be reliable and sensitive to change [40,41]. NRS scales have demonstrated greater levels of sensitivity and discriminatory power than VRS scales [42]. In addition the standardised format with which NRS scales are applied across cultures and languages (typically an 11-point 0-10 scale) means that patients are familiar with this manner of assessment; as opposed to formulations of VRS scales which can often vary in the number of designated response options and the labels assigned to these response options [42]. Patient familiarity of NRS response scales is also reflected in patient estimates of meaningful differences on such scales (2-3 points) which are not only similar for ratings of average pain and worst pain in this study but also evaluations of clinically meaningful difference on 0-10 NRS pain severity measures implemented in other conditions [43]. Published recommendations for core outcome measures in clinical chronic pain trials also support the use of 0-10 NRS scales for assessing pain severity [16].

One limitation, attributable to both the MPQ-SF and BPI-SF is that they do not provide opportunity for patients to differentiate the different forms of pain that they may experience. Feedback from the patients interviewed suggested that they were able to distinguish bone pain from other types of pain such as that associated with CIPN based on the location and nature of this pain, however it is not clear when completing an overall pain assessment whether patients would take into account only the pain associated with bone metastases or all types of pain experienced. There is a chance therefore, that in the context of a clinical trial, improvements in patients' experiences of bone pain may be "washed out" by accompanying neurotoxic side-effects such as CIPN.

Nonetheless, however, from a content validity perspective, evidence supports the use of the BPI-SF 'Worst Pain' item as the most appropriate measure of pain severity in CRPC patients. In addition to demonstrating acceptable content validity in patients with CRPC, a review of evidence in relation to the BPI-SF 'Worst Pain' item also suggests that this item fulfils much of the key criteria specified in the recent FDA PRO Guidance for Industry [24,44]. In particular, there is a wealth of evidence supporting the psychometric validity of this item [45-48]. Further research, however, is needed to confirm these measurement properties within patients with CRPC and bone metastases.

## Conclusions

Among the constellation of symptoms experienced by CRPC patients with bone metastases, bone pain is of key concern. There is, therefore, the need for a reliable and valid measure of pain severity within this population. To this end, the BPI-SF Worst Pain item has demonstrated strong content validity in these patients and, whilst psychometric validity of this item in CRPC patients is to be confirmed, there is considerable evidence from comparable disease areas to support the psychometric properties of this item. A culmination of available evidence suggests, therefore, that the BPI-SF 'Worst Pain' item is an appropriate measure of pain severity for use as a clinical trial endpoint for evaluating treatment efficacy in patients with CRPC and bone metastases.

## Abbreviations

ASR: Age-standardised incidence rate; BPI-SF: Brief Pain Inventory Short-Form; BTcP: Breakthrough cancer pain; CIPN: Chemotherapy-induced peripheral neuropathy; CRPC: Castration-resistant prostate cancer; FDA: Food and Drug Administration; HRQL: Health-related quality of life; IMMPACT: Initiative on Methods, Measurement, and Pain Assessment in Clinical Trial; MPQ-SF: McGill Pain Questionnaire Short-Form; NRS: Numerical rating scale; PPI: Present pain intensity; PRO: Patient reported outcome; VRS: Verbal rating scale.

## Competing interests

Astrazeneca has commissioned Mapi Values, a health outcomes agency with specialist experienced personnel in the field of patient-reported outcomes, to conduct, analyse and communicate findings from this research on their behalf. AG, LA and EP have no other competing interests to declare. CB, BP, SM and FN were all employees of Astrazeneca at the time of the study.

## Authors' contributions

ECP designed the study and led the conduct of the qualitative patient interviews and analysis. BP participated in the design of the study and reviewed study findings. AG conducted analysis of the study findings and developed the manuscript. LA, CB, SM and FN were involved in interpretation of study findings and critical review of the manuscript. All authors read and approved the final manuscript.
